# Factors affecting students’ attitudes towards reproductive health in the north of Iran: Designing an educational program

**DOI:** 10.1186/s12889-023-16217-2

**Published:** 2023-08-16

**Authors:** Afsaneh Bakhtiari, Hajar Pasha, Fatemeh Kashefi, Fatemeh Nasiri-Amiri, Fatemeh Bakouei

**Affiliations:** 1 Social Determinants of Health Research Center, Health Research Institute, Babol University of Medical Sciences, Babol, Iran; 2 Population, Family and Spiritual Research Core, Health Research Institute, Babol University of Sciences, Babol, Islamic Republic of Iran; 3 Student Research Committee, Babol University of Medical Sciences, Babol, Iran; 4 Department of Midwifery and Reproductive Health, Faculty of Nursing & Midwifery, Babol University of Medical Sciences, Babol, Islamic Republic of Iran; 5 Infertility and Reproductive Health Research Center, Health Research Institute, Babol University of Medical Sciences, Babol, Islamic Republic of Iran

**Keywords:** Attitude, Reproductive Health, Educational Models, James Brown mode, Young Student

## Abstract

**Background:**

It is essential to empower young people to promote reproductive health (RH) and develop effective educational programs to prevent risky behaviors. This study aimed to investigate the factors affecting students’ attitudes towards RH based on the ecological model and then design an educational program.

**Methods:**

This cross-sectional study was conducted on 461 female students aged 18–29 in the north of Iran. This study was done in two stages. In the first stage, factors affecting the attitude towards RH including demographic questionnaire, interpersonal communication skills, family communication pattern, depression, stress and anxiety, body self-image, and self-confidence were determined. In the second stage, an educational program was designed based on the most effective factors. Independent t-test, ANOVA, and multiple linear regression were employed using SPSS version 20 software. Also, STATA version 15 software was utilized for statistical modeling to predict the best predictive model of attitude towards RH.

**Results:**

47.7% of students had a good attitude toward RH. The majority of students had problems with interpersonal communication skills (60.7%). Also, 28.5% experienced depression, 35.8% anxiety, and 12.8% stress at different levels. More than a quarter of the students (26.5%) had poor body self-image and 18.7% had Undesirable self-esteem. Interpersonal skills (P = 0.002), family communication pattern (P = 0.004), stress (p = 0.019), anxiety (P = 0.001), and body self-image (P = 0.034) have a significant relationship with the attitude towards RH. The multiple regression showed that the most important effective factor on RH is the dialogue orientation of family communication pattern (P = 0.041), stress (P = 0.002), and anxiety (P = 0.001).

**Conclusion:**

Stress and anxiety management training and the use of dialogue orientation in the family communication pattern for young female students are recommended based on the scientific model.

**Supplementary Information:**

The online version contains supplementary material available at 10.1186/s12889-023-16217-2.

## Introduction

Today, reproductive health (RH) has been classified under reproductive rights, and its expansion as a guarantee for the health of society and the development of countries depends on the health of young people [1]. During youth, important behavior patterns are formed, including reproductive and sexual attitudes and behaviors [2]. The RH status of young people is a major health issue in any society [1], which has received less attention in our society because it has been regarded as a taboo. Many young people in Iran, regardless of gender, do not benefit from sufficient opportunities to improve their overall health, including RH [2, 3]. While the government has reaffirmed many commitments for young people’s wellbeing such as access to SRH services, policy development falls far short of realizing these commitments [4]. Passing from youth to adulthood without sufficient knowledge and a positive attitude about reproductive and sexual health information and services causes repercussions [5]. As a high-risk group, young people are exposed to smoking, alcohol and drug addiction, sexually transmitted diseases and AIDS, unsuccessful marriages and divorces, infertility, unwanted pregnancies, unsafe abortions, and maternal mortality [6].

Nowadays, with the rapid development of technology and easy access to information sources in the world, young people are more than ever at risk of the influx of false information, especially in the field of RH, which will have a negative impact on their attitudes [7].The existence of a negative attitude toward RH can cause harmful behaviors in young people [8]. Attitude is the first determining factor of behavioral intention, and the more favorable the attitude is toward the behavior, the stronger the individual’s intention is for that conduct [9]. Several intervention programs have been designed to improve the attitude and optimal performance of RH among young people in the world, and a few of them have been successful [4, 10–12]. It is believed that the reason for this lack of success is the scarcity of studies on the factors affecting the attitudes and beliefs of young people in the area of RH issues. Various hypotheses have been proposed to explain a potential relationship between attitudes and risky sexual behavior. The ecological theory argues that risky sexual behavior among the youth is primarily influenced by three interrelated systems of Microsystem, Mesosystem and Macrosystem [13, 14]. Microsystem includes age, gender, race, marital status, residence place, religion, education level, personality traits, psychological problems, childhood experiences, and body image. The mesosystem comprises the factors such as family structure, relationships with others, and sex education. In the macrosystem, the influence of society’s culture and tradition, economic situation and media are presented [15]. These factors can reliably help us identify the risk and protective factors for the physical and mental health of young people [14].

Several studies have been conducted on the status of young people’s attitudes about RH in different countries [8, 16–18] and Iran [2, 13, 19], which mostly investigated knowledge, attitude and performance of young people towards RH. However, few studies have been carried out on the factors affecting young people’s attitudes about RH from an ecological point of view and at the same time designing an educational intervention program based on the most effective factor. It is necessary to comprehensively identify the factors affecting young people’s attitudes towards RH to design educational content that fits the needs of young people’s RH. This is to improve their performance and can lead to the correct implementation of preventive interventions based on the real needs of young people. Considering the importance of the young generation in the development of nations, the present study determines the factors affecting RH attitudes in the first step, and in the second step, designs an educational program based on the most effective factors on RH attitudes.

## Methods

### Study design

This cross-sectional study was conducted in 2018 on the students of public universities (including the University of Technical Engineering, Medical Sciences and Human Sciences) in Babol city, Mazandaran province, Iran. Public universities are funded and regulated by the Iranian government, unlike private universities that are funded by private organizations.

The current study adapted the theoretical framework of the ecological model in the selection of the variables used for analysis [13, 14]. Accordingly, in this study, the microsystem and mesosystem were regarded as psychological factors (depression, anxiety, stress, body self-image and self-esteem) and communication factors (interpersonal communication and family communication pattern) respectively. The results of the macrosystem variables and their relationship with some other factors were published elsewhere [20]. For data collection, each university was visited separately daily. In each university, students’ meeting places including classrooms, self-service, mosques, dormitories, and sports halls were first referred to, and after a full introduction of the topic, purpose and method of the research, the students were invited to cooperate. In the case of the desire and eligibility criteria, the written consent form was completed by the participants and then the questionnaires were distributed among them.

The inclusion criteria consisted of age 18–29 years (according to the age guidelines of the Ministry of Health; integrated care plan) [21], female sex, single at the time of the study, willingness and informed consent to enter the research. The exclusion criteria were failure to answer more than 10% of the questionnaire questions.

### Sample size

The sample size required to determine the relationship between the factors affecting the attitude to RH at the confidence level of 95% and the test power of 80% to identify the relationship of r = 0.15 was 400 people(Using G power software), which was calculated with a 15% sample loss of 460 people. For sampling, each university was considered one class. Then, the samples in each class were extracted by random sampling from among the eligible students based on the allocated percentage of the total population required for that class. In this way, after taking into account the number of quotas of each university compared to the total number of single female students 18 to 29 years old studying in that university, 132 samples from Medical Sciences University, 175 samples from Technical and Engineering University and 154 samples from Payam Noor University were included in the study.

### Measures

The data collection tools were demographic characteristics (age, residence place, educational level, parents’ education and occupation, family income from the individual’s point of view), RH Attitude Questionnaire [20, 22, 23], Interpersonal Communication Skills Test [24], Revised Family Communication Pattern(RFCP) [25], Depression, Anxiety, Stress Scale(DASS) [26], Body Image Acceptance and Action Questionnaire (BI-AAQ)[27], and Rosenberg Self-Esteem Questionnaire[28]. The data collection instruments are shown in Supplementary Material file1.

The average time to answer the questions was 20 min, and during this time the researcher answered the students’ questions. After collecting the questionnaires, prizes were distributed among the students as a token of appreciation.

### Statistical analysis

The data were analyzed using SPSS version 20 software as well as STATA version 15 software for statistical modeling to predict the best predictive model of attitude towards RH. Examining the normality of the data using the Kolmogorov-Smirnov test and histogram showed that the data were normal. To analyze the data, descriptive statistics methods (mean standard deviation, relative frequency and percentage) and then inferential tests such as independent t-test and ANOVA were used. Also, multiple linear regression using STATA software examined the relationship between psychological (DASS, body image, self-esteem) and communication (family communication pattern Interpersonal communication skills) variables with RH attitude. Also for modeling and selecting the best variables in the regression model, R2 adjust, Mallow cp., and AIC indices were used. Finally, the best model of psychological and communication variables were selected separately. Also to check model defaults, the histogram and Q-Q plot were employed to check the normality of the residuals. Breusch-pagan/cook-Weisberg test was applied to test the homogeneity of error variance in the regression model.

## Educational design by James Brown’s method

To design the educational program after determining the most effective factor on RH attitude, James Brown’s regular teaching pattern was adopted (Table 1). The most comprehensive definition of educational technology is provided by James Brown. The model has five parts: identify needs and educational goals (in three parts, general, specific and behavioral goals), educational conditions (in three parts of learning experiences, teaching and learning groups, and teaching methods), educational resources (in three parts of human resources, educational tools and materials, and educational spaces) and efficiency including evaluation and correction [29]. In the central core of this model lies the student element which shows attention to interests, talents and other student characteristics as the primary element of education [30]. Educational design by the James Brown method has been long used by teachers and researchers and has brought positive and successful results [31, 32]. Researchers have used this educational design model in reproductive and sexual health education and also to change the attitude of the target group [29–31].

## Lesson plan (materials and methods)

After introducing and stating the goals, the pre-test sheets will be distributed to the students to complete it. The students who prefer anonymity on the pre-test sheet will enter a five-letter code of their choice on the top and left side of the pre-test sheet and will remember the desired code for the post-test sheet. After ten minutes, the sheets will be collected. Then the educational content will be taught by the teacher cooperatively and dynamically according to the general, specific and behavioral goals considered in the lesson plan. At the end of each topic, students will be allowed to ask questions.

At the end of the training course, the students who are actively present in all the training hours and obtain the quorum score in the post-test will be given a training certificate. To consolidate the teachings, homework was provided in the form of files and CDs, exercises with the worksheets will be provided in the class and an introduction of the book to the workshop participants. The students will study the educational resources for one month and will use the breathing and relaxation techniques learned in the workshop based on the educational resources provided to them in times of stress and anxiety. Then one month after the workshop, the participants will be provided with the post-test sheet. After 10 min, the sheets will be collected after the student’s code or surname was entered.

In the efficiency section, educational results will be initially evaluated. When correction was required, educational redesign will be performed to solve the educational problem by reviewing all the stages of the model.

**Table 1:** Designing an educational program based on the James Brown model for the main variables affecting the attitude to reproductive health

**General goals**	The first goal - Stress variable: familiarity with stress, symptoms and effects of stress, stress management methods (first session - time: 180 min)		
	The second goal- Anxiety variable: familiarity with anxiety and fear Management (second session- time: 180 min)		
	The third goal- variable dialogue in family communication pattern: familiarity with family types, family communication pattern, dialogue orientation and its importance in family communication pattern (third session- time: 90 min)		
**Specific goals**	**Student can**: 1. Define stress. 2. Explain the types of stress. 3. Express the difference between a pleasant and unpleasant stress. 4. Explain stress signs. 5. Explain the stress effect. 6. Express the importance of dealing with stress. 7. Express the physical and psychological effects of stress. 8. Describe the stressors. 9. Mention the types of stress prevention strategies. 10. Describe the effect of spirituality in reducing stress. 11 Provide the methods of dealing with stress. 12. Implement one of the ways to deal with stress. 13. Express relaxation methods. 14. Implement one of the methods of relaxation.	**Student can**: 1- Define anxiety. 2. Define fear. 3. Mention the difference between fear and anxiety. 4. Explain the signs and symptoms of anxiety. 5. Express the cause of anxiety. 6. Express effective factors in reducing anxiety. 7. State the relationship between stress and anxiety. 8. Express the importance of dealing with anxiety. 9. Express anxiety management ways. 10. Implement one of the methods of managing anxiety.	**Student can**: 1. Define the family. 2. Explain the types of family from the perspective of the communication pattern. 3. Define communication skill. 4- Express the types of orientation in the family communication pattern. 5. Explain the conversation of the family in the family communication pattern. 6. The importance of the impact of the pattern of dialogue on the positive attitudes and appropriate behavior of the children. 7. Explain the alignment orientation in the family communication pattern. 8. The impact of the dialogue orientation on the development of personality traits and the development of children in society. 9. The impact of family communication styles on the development of personality traits and children’s development.
**Behavioral goals**	**Student can be in the presence of a lecturer without any educational source**: 1. Define stress in a line (cognitive). 2. Fully express the types of stress (cognitive). 3. Express the difference between pleasant and unpleasant stress in two lines (cognitive). 4. Briefly explain all the symptoms of stress (cognitive). 5. Briefly explain the stress effects (cognitive). 6. Express the importance of dealing with stress in three lines (emotional). 7. Briefly state the physical and mental effects of stress (cognitive). 8. Explain the stressors (cognitive). 9. Summarize the types of stress prevention strategies (cognitive). 10. The effects of spirituality on reducing stress in three lines (cognitive). 11. Summarize the ways to deal with stress (cognitive). 12. Perform one of the ways to deal with stress (psycho -motor). 13. Briefly state the different methods of relaxation (cognitive). 14. Perform one of the methods of relaxation (psycho -motor).	**Student can be in the presence of a lecturer without any educational source**: 1- Define anxiety completely (cognitive). 2. Define fear (cognitive). 3. Mention the difference between fear and anxiety in two lines (cognitive). 4. Explain the main signs and symptoms of anxiety (cognitive). 5. Summarize the cause of anxiety (cognitive). 6. Express the effective factors in reducing anxiety (cognitive). 7. Express the relationship between stress and anxiety in two lines (cognitive). 8. Summarize the ways of managing anxiety (cognitive). 9. Express the importance of dealing with anxiety in three lines (emotional). 10. Perform one way of managing anxiety completely (psycho-motor).	**Student can be in the presence of a lecturer without any educational source**: 1. Define the family in a line (cognitive). 2- Briefly explain the types of family from the perspective of the communication pattern (cognitive). 3. Define communication skills in one line (cognitive). 4. Summarize the orientation in the family communication pattern (cognitive). 5. Explain the dialogue orientation in the family communication pattern (cognitive). 6. The importance of the impact of the dialogue pattern on the positive attitude and appropriate behavior of children (emotional). 7. Explain the orientation of synchronization in the family communication pattern (cognitive). 8. Describe the impact of the orientation of the dialogue on the development of the personality traits and the development of children in social affairs with an example (cognitive). 9. Summary the impact of family communication styles on the development of personality traits and development of children (cognitive).
**Conditions**			
**Learning experiences**	Active Listening to educational content, participation in group discussion, answering questions raised, executing activities requested at the meeting, studying at home, doing assignments at home		
**Teaching and learning groups**	Groups of 15–25		
**Teaching methods**	Programmed lecture, lecture, group discussion, colloquy, movie show, role play		
**References**			
**Human resources**	According to a survey of the students, one or a combination of the following human resources will be considered: a psychiatrist, a trained university lecturer, trained expert		
**Educational material**	Video Projector, Computer, Whiteboard and Magic in different colors, colors, stress management tutorials, training slides, stress management training video, work worksheets requested at home, pre -test and post -test sheets		
**Educational spaces**	Suitable educational spaces at the university such as student meeting halls, classrooms, etc. are based on existing conditions and audience surveys.		
**Evaluation**			
**Step Evaluation**	Step evaluation is done using question & answer, feedback from learners, practical activities and post -test participation at the end of the training session.		
**Final evaluation**	One month after the end of the sessions, it is done by examining the educational materials learned and the activities carried out by the student.		
**The evaluation modification**	Based on the results of the evaluation of the previous steps, the modification of the educational components is done.		

## Results

Out of the 461 respondents, the majority (62.3%) were between 18 and 21 years old .The majorities (83.3%) were an undergraduate and general practitioner. The demographic characteristics are shown in Table 2. The mean score of attitudes toward RH was 58.34 ± 7.05 with a minimum score of 38 and a maximum of 80. Moreover, 47.7% of the students had a good attitude toward RH.

**Table 2:** Demographic characteristics of the participants

Variables	Frequency	Percent	Variables	Frequency	Percent
**Educational Level**			**Mothers Education**		
BSc/GP/MSc	384	83.3	High school ≤	307	66.6
PhD	77	16.7	University	154	33.4
**Study Field**			Fathers Education		
Medicine/par medicine	132	28.6	High school ≤	258	56
non-medical	329	71.4	University	203	44
**Age Groups**			Mother’s Occupation		
18–21	287	62.3	homemaker	322	69.8
22–25	149	32.3	employed	63	13.7
26–29	25	5.4	Retired	76	16.5
Family income (individual’s point of view)			Father’s Occupation		
Adequate	349	75.7	jobless	7	1.5
Inadequate	112	24.3	employed	378	82
			Retired	76	16.5

Notably, 60.7% of the students had interpersonal communication problems. The most common family communication pattern (70.3%) was dialogue orientation. Also, 10.7% showed moderate to severe depression, 23% moderate to severe anxiety and 12.8% mild to moderate stress. Almost half of the students (49.5%) had a good/very good body self-image. Finally, 18.7% reported undesirable self-esteem (Table 3).

**Table 3:** The communication and psychological variables of the participants

Variables	Frequency	Percent	Mean ± SD	Min	Max
**Interpersonal communication skills**			61.77 ± 10.12	30	93
Acute communication problem	22	4.8	40.41 ± 4.06	30	45
Communication problem	280	60.7	57.30 ± 4.87	46	65
Capable	159	34.5	72.61 ± 6.06	66	93
**Family communication pattern**					
Dialogue	324	70.3	40.22 ± 9.00	13	59
Alignment	137	29.7	27.64 ± 6.78	7	44
**Depression**			6.80 ± 4.80	00	28
Normal	329	71.4	4.33 ± 2.75	00	9
Mild	82	17.8	11.15 ± 1.10	10	13
Moderate	46	10	15.76 ± 1.89	14	20
Severe	3	0.6	21 ± 0.00	21	27
Very sever	1	0.2	28 ± 0.00	28	28
**Anxiety**			6.25 ± 4.37	00	21
Normal	296	64.2	3.54 ± 2.23	00	7
Mild	59	12.8	8.53 ± 0.50	8	9
Moderate	88	19.1	11.68 ± 1.45	10	14
Sever	16	3.5	16.19 ± 1.22	15	19
Very severe	2	0.4	21 ± 00	20	21
**Stress**			9.33 ± 4.43	00	21
Normal	402	87.2	8.22 ± 3.52	00	14
Mild	43	9.3	15.81 ± 0.98	15	18
Moderate	16	3.5	19.75 ± 0.77	19	25
**Body self- image**			31.78 ± 16.16	12	84
Quite good	123	27	14.63 ± 2.19	12	18
Good	104	22.5	22.69 ± 2.58	19	27
Medium	112	24.3	34.76 ± 4.44	28	42
Weak	122	26.5	54.08 ± 9.20	43	84
**Self-esteem**			10.64 ± 3.82	00	30
Undesirable	82	18.7	19.92 ± 3.13	00	14
Good	307	66.8	28.04 ± 1.53	15	25
Quite good	72	14.5	10.64 ± 3.82	26	30

The association of interpersonal communication with attitude towards RH showed that capable students, compared to individuals with communication problems (P = 0.002), and the students with dialogue orientation in the family, compared to the alignment orientation, showed a more significant attitude (P = 0.004). Among the psychological variables, anxiety (P = 0.001), stress (P = 0.019) and good body self-image (P = 0.034) demonstrated a significant relationship with the attitude towards RH (Table 4).

**Table 4:** The association of communication and psychological variables with the participants’ attitude to reproductive health

Variables	Mean ± SD	P-value
**Interpersonal communication skills**		0.002
Acute communication problem	57.82 ± 5.87	
communication problem	57.49 ± 6.82	
capable	59.92 ± 7.35	
**Family communication pattern**		0.004
dialogue	58.80 ± 7.12	
Alignment	56.74 ± 6.67	
**Depression**		0.197
Normal	58.67 ± 7.21	
Mild	58.13 ± 6.45	
Moderate	56.32 ± 5.78	
Severe	59.66 ± 18.00	
Very Severe	58.67 ± 7.21	
**Anxiety**		0.001
Normal	59.36 ± 6.94	
Mild	56.64 ± 7.09	
Moderate	56.68 ± 6.56	
Severe	56.12 ± 7.27	
Very Severe	50.00 ± 15.55	
**Stress**		0.019
Normal	59.45 ± 7.05	
Mild	57.97 ± 7.08	
Moderate	55.81 ± 7.02	
**Body self- image**		0.034
Quite good	60.00 ± 6.72	
Good	58.14 ± 6.84	
Medium	58.16 ± 6.93	
Weak	57.30 ± 7.45	
**Self-esteem**		0.116
Undesirable	57.43 ± 7.64	
Good	58.26 ± 6.98	
Quite good	59.76 ± 6.49	

Anova and T-test

The results of multiple linear regression analysis to determine the effective factors on RH attitude revealed that according to the determination coefficient of regression analysis, communication variables 5.3%, psychological variables 8.5% and the simultaneous presence of communication and psychological variables explained 10% of changes in RH attitude (Table 5).

**Table 5:** The relationship between communication and psychological variables with attitudes towards reproductive health

Variables	Standardized Coefficients Beta	CI (95%)	P value
Communication variables			
Interpersonal communication	0.073	0.008, 0.138	0.027
Dialogue	0.078	0.019, 0.137	0.010
Alignment	-0.018	-0.094, 0.056	0.628
**R**^**2**^ **= 0.053**			
**Psychological variables**			
Depression	-0.13	-0.331, 0.066	0.191
Anxiety	-0.42	-0.624, -0.229	0.001
stress	-0.32	-0.125, 0.525	0.001
Body self-image	0.23	-0.002, 0.067	0.295
Self-esteem	0.129	0.001, 0.256	0.047
**R**^**2**^ **= 0.085**			
**Communication & Psychological variables**			
Interpersonal communication	0.04	-0.019, 0.117	0.163
Dialogue	0.06	0.002, 0.124	0.041
Alignment	-0.003	-0.080, 0.072	0.919
Depression	-0.08	-0.289, 0.111	0.384
Anxiety	-0.41	-0.613, -0.220	0.001
stress	-0.31	0.111, 0.510	0.002
Body self- image	0.02	-0.019, 0.068	0.273
Self-esteem	0.06	-0.070, 0.199	0.348
**R**^**2**^ **= 0.100**

-The results are adjusted for age, self-religion, conversation with parents about RH concepts, living with parents, use of satellite, smoking, alcohol, and participation in youth parties.
-Multiple linear regression analysis.

The major factor affecting students’ RH attitude was stress, anxiety and family communication pattern as found by multiple linear regression. Therefore, the educational design was made based on James Brown’s systematic educational model. Based on this, the students participate in a three-day workshop with three educational topics: stress management for 3 h, anxiety control for 3 h, and familiarization with dialogue orientation in family communication for 1.5 h. The pre-test and post-test are conducted using DASS and RFCP questionnaires before and one month after the workshop. The training program is shown in Table 1 and Supplementary Material file2.

## Discussion

The study results to determine the influencing factors on the attitude of young students towards RH revealed that ability in interpersonal communication, as well as the dialogue orientation in family communication, was associated with a better attitude towards RH. Also, among the psychological variables, anxiety, stress and good body self-image had a significant relationship with the attitude of RH. In the present study, an ecological approach was adopted to achieve a more complete understanding of the factors affecting attitude; an approach that demonstrates the interaction between multiple levels of health outcomes. Researchers rarely move beyond individualistic approaches in terms of attitude to RH [32]. The present study is unique in analyzing the factors affecting the attitude of young people on RH with a systemic approach.

Our study showed that individuals capable of interpersonal communications had a better attitude to RH than people with communication problems. Effective interpersonal communication enhances physical and mental health and improves adverse attitudes and risky behaviors. Also, self-esteem and the ability of individuals to deal with life’s problems are largely influenced by interpersonal communication [33]. A study of Iranian students showed that 57.3% of them had a communication problem and 0.6% had an acute communication problem. Therefore, the researchers found it necessary to hold educational workshops to improve students’ communication skills [34]. Some studies mentioned peer pressure and interpersonal communication as the most important factor in risky behaviors in teenagers [35, 36]. A study on the importance of interpersonal communications in American black students showed that interpersonal health strategies are essential along with sexual behavior training to make changes in sexual beliefs and behaviors [37]. However, other studies have shown that interpersonal communications reinforce the effects of intervention after being exposed to sexual health interventions [38, 39]. A systematic meta-analysis of 28 studies has shown that mass media can help to some extent in understanding the norms associated with a behavior, but conversations with others provide a more detailed understanding of the normative values of a behavior that can influence attitudes, intentions, or behavior [40]. Brindis et al. (2020) in the United States proposed a multi-faceted approach to reducing pregnancy in adolescents, including radio, theater, interpersonal communication and inter-generational dialogue [41]. A study in Nepal also showed that interventions based on dialogue were efficacious in changing behavior and preventing abortion in the country [42]. Therefore, upgrading interpersonal communication skills in young people plays an important role in preventing high-risk behaviors.

The present study also revealed that the family’s most common communication pattern was the dialogue pattern, and the students in these families had a better attitude to RH. It is well known that family characteristics and communication between its members play an important role in preventing high-risk behaviors and mental health of young people [43, 44]. The family environment is the best place for the emotional and social development of children and has the greatest impact on how the young behave and understand themselves. Members of high-dialogue families interact freely and constantly and make collective decisions. In such families, children consult their parents instead of searching information in magazines and websites and gaining information from peers [25, 45]. The studies showed that dialogue orientation in the family communication pattern directly improves students’ ability to adapt to needs and tackle stressors to achieve academic and social success [46–48]. Studies also showed the effects of good family communication on improving the quality of young people’s life [45], reduced the likelihood of sexual intercourse in girls [49], personality traits and reduced anxiety and depression in youth [50].

The present study focused on the relationship between stress and anxiety with the attitude of RH. There are few studies on the impact of psychological factors (depression, stress, and anxiety) on student RH attitudes. A study in Bosnia and Herzegovina indicated a high prevalence of stress among students. Therefore, the researchers considered stress management and mental health interventions necessary for students [51]. Another study on young Mexican American women showed that individual factors such as stress, anxiety, self-efficacy, beliefs, family factors (parental relationship with children and educating children on sexual issues), and peers impact the start or delay of sexual intercourse [52]. The study of Karle et al. showed that poor mental health was associated with increasing high-risk sexual behavior in Swedish youth [15]. Maintaining mental health is critical for safer sexual behavior in young people. Studies around the world have found a relationship between psychological symptoms and high-risk sexual behaviors among young people [53–55]. Accordingly, identifying important sources of stress and anxiety in young people and their control methods can play an important role in improving the attitude of RH.

Self-esteem and body self-image also play an important role in young people’s health. The negative perception of one’s body image reduces self-esteem, which in turn increases psychological distress. Self-esteem is a major predictor in young people that affect interpersonal relationships and academic performance. On the contrary, low self-esteem affects aggression, antisocial behavior, youth delinquency, and negative changes in body image [56]. The body self-image is a predictor of the mental health of young people that can influence healthy or unhealthy behaviors because of its relationship with the physical, cognitive and emotional dimensions of people. A positive body self-image is can prevent health problems in students [57]. A study on Romanian students also showed that body dissatisfaction in women depends on the level of self-esteem [56]. Lin et al. study (2018) on students at Taiwan University also showed that body self-image and self-esteem are the main predictors of sexual satisfaction [58]. The relationship between self-esteem and the general health of the students [59], as an indicator of transactional sex in young African women [55], Pakistani students’ academic performance [60], addiction and pathological use of the Internet and Social Media and life satisfaction in students have been well investigated [61].

Reproductive-sexual health is central in education, has an important role in the formation of human personality, and has an impact on their thoughts, emotions, attitudes and behaviors of young people [2]. Many factors can affect young people’s attitude to RH, mainly communication and psychological factors. Investigating the factors affecting youth attitudes on RH helps identify the risks and factors in the physical and mental health of youth [15]. Scientific and community-based perspectives can improve performance in youth RH and can properly implement preventive interventions based on the real needs of the youth [11].

### Limitations and Strengths

A limitation of this study is the use of a self-reporting questionnaire. Young people typically avoid discussing sex issues. Therefore, answers to the sex-related questions are commonly vague. Data were also collected only from female students and the results may not be generalized to all young people. Strength of this study is the employment of an ecological model in examining the factors affecting RH. It is also possible to mention the acceptable sample size, the detailed design and implementation of the study and finally the presentation of an educational program based on the James Brown model.

## Conclusion

The results of this study showed that stress, anxiety and family communication patterns are major factors affecting the attitude of RH in young female students. Given that adverse attitudes toward RH can expose young people to irreversible psychological and physiological damages, designing educational programs to improve interpersonal communications and control stress and anxiety based on scientific models can raise awareness and create a favorable attitude in RH to prevent the consequences of adverse attitudes and high-risk behaviors in young people. Finally, parental education, especially on how to communicate and provide information to young people, should not be overlooked.

### Supplementary Information

**Additional file 1.** Data collection instruments.

**Additional file 2.** Appendix.

## Data Availability

Data about individual identified samples of this research will be available from the corresponding author on reasonable request after the main results of the research have been published.

## Declarations

**Ethics approval and consent to participate**.

This study was confirmed by the ethics committee of the Babol University of Medical Sciences with the ethical code IR.MUBABOL.HRI.REC.1397.129. Informed consent was obtained from all subjects before the research commenced. The research was conducted according to the declaration of Helsinki.

**Consent for publication**.

## References

[1] Benyamini Y, Todorova I (2017). Women’s reproductive health in sociocultural context. Int J Behav Med.

[2] Farahani FK (2020). Adolescents and young people’s sexual and reproductive health in Iran: a conceptual review. J Sex Res.

[3] Mohammadi F, Kohan S, Mostafavi F, Gholami A (2016). The Stigma of Reproductive Health Services utilization by unmarried women. Iran Red Crescent Med J.

[4] Khalajabadi Farahani F, Darabi F, Yaseri M. The Effect of theory-based HIV/AIDS educational program on preventive behaviors among female adolescents in tehran: a Randomized Controlled Trial. 2020;21(3):194–206. PMC7362097 32685417

[5] Beymer M, Willmes CG (2021). Fruitful futures for reproductive health. Trends Endocrinol Metab.

[6] Swartzendruber A, Brown JL, Sales JM, Windle M, Haardörfer R (2019). Age-related associations between substance use and sexual risk behavior among high-risk young african american women in the South. Addict Behav.

[7] Gollub EL, Beauvais S, Roye C (2022). College-attending young men’s sexual and reproductive health knowledge, attitudes and practices. J Am Coll Health.

[8] Zakaria M, Karim F, Mazumder S, Cheng F, Xu J (2020). Knowledge on, attitude towards, and practice of sexual and reproductive health among older adolescent girls in Bangladesh: an Institution-Based cross-sectional study. Int J Environ Res Public Health.

[9] Howe LC, Krosnick JA (2017). Attitude strength. Annu Rev Psychol.

[10] Darabi F, Yaseri M, Kaveh MH, Khalajabadi Farahani F, Majlessi F, Shojaeizadeh D (2017). The Effect of a theory of planned behavior-based educational intervention on sexual and reproductive health in iranian adolescent girls: a Randomized Controlled Trial. J Res Health Sci.

[11] Denno DM, Hoopes AJ, Chandra-Mouli V (2015). Effective strategies to provide adolescent sexual and reproductive health services and to increase demand and community support. J Adolesc Health.

[12] Desrosiers A, Betancourt T, Kergoat Y, Servilli C, Say L, Kobeissi L (2020). A systematic review of sexual and reproductive health interventions for young people in humanitarian and lower-and-middle-income country settings. BMC Public Health.

[13] Arabi-Mianrood H, Hamzehgardeshi Z, Khoori E, Moosazadeh M, Shahhosseini Z. Influencing factors on high-risk sexual behaviors in young people: an ecological perspective. Int J Adolesc Med Health. 2017 19;31(2):j/ijamh.2019.31.issue-2/ijamh-2016-0162/ijamh-2016-0162.xml. 10.1515/ijamh-2016-0162 28422704

[14] Khuzwayo N, Taylor M (2018). Exploring the socio-ecological levels for prevention of sexual risk behaviours of the youth in uMgungundlovu District Municipality, KwaZulu-Natal. Afr J Prim Health Care Fam Med.

[15] Karle A, Agardh A, Larsson M, Arunda MO (2023). Risky sexual behavior and self-rated mental health among young adults in Skåne, Sweden - a cross-sectional study. BMC Public Health.

[16] Kilfoyle KA, Vitko M, O’Conor R, Bailey SC (2016). Health literacy and women’s reproductive health: a systematic review. J Womens Health (Larchmt).

[17] Mazza D, Botfield JR (2022). Sexual and reproductive health. Semin Reprod Med.

[18] Zou S, Cao W, Jia Y, Wang Z, Qi X, Shen J, Tang K (2022). Sexual and reproductive health and attitudes towards sex of young adults in China. BMJ Sex Reprod Health.

[19] Honarvar B, Salehi F, Barfi R, Asadi Z, Honarvar H, Odoomi N, Arefi N, Lankarani KB (2016). Attitudes toward and experience of singles with premarital sex: a Population-Based study in Shiraz, Southern Iran. Arch Sex Behav.

[20] Kashefi F, Bakhtiari A, Pasha H, Amiri FN, Bakouei F (2021). Student attitudes about reproductive health in public universities: a cross-sectional study. Int Q Community Health Educ.

[21] World Health Organization. Regional Office for the Eastern Mediterranean. Minister of Health and Medical Education. Islamic Republic of Iran health profile 2015. https://apps.who.int/iris/handle/10665/253768?show=full.

[22] Cleland J, Ingham R, Stone N (2001). Asking young people about sexual and reproductive behaviours: illustrative core instruments. Occasional Report No 13. Human Reproduction.

[23] Olfati F, Aligholi S (2008). A study on educational needs of teenager girls regarding the reproductive health and determination of proper strategies in achieving the target goals in Qazvin. J Inflamm Dis.

[24] Vakili MM, Hidarnia AR, Niknami S (2012). Development and psychometrics of an interpersonal communication skills scale (A.S.M.A) among Zanjan health volunteers. J Hayat.

[25] Hassanzadeh M (2013). Investigation of the relationship between family communication patterns and social creativity in students. IJBS.

[26] Kakemam E, Navvabi E, Albelbeisi AH, Saeedikia F, Rouhi A, Majidi S (2022). Psychometric properties of the Persian version of depression anxiety stress Scale-21 items (DASS-21) in a sample of health professionals: a cross-sectional study. BMC Health Serv Res.

[27] Izaadi A, Karimi J, Rahmani M (2013). Psychometric analysis of persian version of body image flexibility questionnaire (BI-AAQ) among University students. J Hayat.

[28] Shapurian R, Hojat M, Nayerahmadi H (1987). Psychometric characteristics and dimensionality of a persian version of Rosenberg Self-esteem scale. Percept Mot Skills.

[29] Khodakarami B, Aligholi S (2011). The Effect of education based on James Brown Pattern on Knowledge of Reproductive & sexual health in women participant marriage council classes in Hamadan. Avicenna J Nurs Midwifery Care.

[30] Yosefi Z, Afshar M, Mirbagher Ajorpaz N, Sadat Z (2021). The Effect of training based on James Brown Model on self-efficacy in adolescents with type 1 diabetes mellitus. J Holist Nurs Midwifery.

[31] Salazar LF, Bradley EL, Younge SN, Daluga NA, Crosby RA, Lang DL, DiClemente RJ (2010). Applying ecological perspectives to adolescent sexual health in the United States: rhetoric or reality?. Health Educ Res.

[32] Abedini S, Nooraddin S, Ghanbarnejad A (2015). The effectiveness of educational programs based on james brown model on knowledge, attitudes and behaviors related to nutritional iron deficiency anemia in female high school students. J Chem Pharm Res.

[33] Munro-Kramer ML, Skidmore LM, Cannon LM, Huhman AK, Carter SN, Williamsen KM, Ngo QM (2022). The Dynamics of interpersonal relationships: understanding power and control tactics among college students. J Interpers Violence.

[34] Namazi A, Homauonfar H (2017). Assessment of interpersonal communication skills and related factors in nursing and midwifery students. Health_Based Res.

[35] Cherie A, Berhane Y (2012). Peer pressure is the prime driver of risky sexual behaviors among school adolescents in Addis Ababa, Ethiopia. World J AIDS.

[36] Srahbzu M, Tirfeneh E (2020). Risky sexual behavior and associated factors among adolescents aged 15–19 years at governmental high schools in Aksum Town, Tigray, Ethiopia, 2019: an Institution-Based, cross-sectional study. Biomed Res Int.

[37] Francis DB, Zelaya CM, Fortune DA, Noar SM (2021). Black college women’s interpersonal communication in response to a sexual health intervention: a mixed methods study. Health Commun.

[38] Helme DW, Noar SM, Allard S, Zimmerman RS, Palmgreen P, McClanahan KJ (2011). In-depth investigation of interpersonal discussions in response to a safer sex mass media campaign. Health Commun.

[39] Frank LB, Chatterjee JS, Chaudhuri ST, Lapsansky C, Bhanot A, Murphy ST (2012). Conversation and compliance: role of interpersonal discussion and social norms in public communication campaigns. J Health Commun.

[40] Jeong M, Bae RE (2018). The effect of campaign-generated interpersonal communication on campaign-targeted Health Outcomes: a Meta-analysis. Health Commun.

[41] Brindis CD, Decker MJ, Gutmann-Gonzalez A, Berglas NF (2020). Perspectives on adolescent pregnancy prevention strategies in the United States: looking back, looking Forward. Adolesc Health Med Ther.

[42] Bingham A, Drake JK, Goodyear L, Gopinath CY, Kaufman A, Bhattarai S (2011). The role of interpersonal communication in preventing unsafe abortion in communities: the dialogues for life project in Nepal. J Health Commun.

[43] Farahani FK, Cleland J, Mehryar AH (2011). Associations between family factors and premarital heterosexual relationships among female college students in Tehran. Int Perspect Sex Reprod Health.

[44] Muhwezi WW, Katahoire AR, Banura C, Mugooda H, Kwesiga D, Bastien S, Klepp KI (2015). Perceptions and experiences of adolescents, parents and school administrators regarding adolescent-parent communication on sexual and reproductive health issues in urban and rural Uganda. Reprod Health.

[45] Mashalpoure Fard M (2020). The relationship between family communication patterns and adjustment with resiliency in children. J Res Health.

[46] Ghaffari M, Esmali A, Mohammadi R (2021). The mediating role of family communication patterns in the relationship between emotional maturity and self-compassion with vulnerability to stress in soldiers. JPMed.

[47] Dorrance Hall E, Scharp K, Sanders M, Beaty L (2020). Family communication patterns and the mediating effects of support and resilience on students’ concerns about college. Fam Relat.

[48] Ehyakonandeh M, Yousefi F (2017). Relationship between family communication patterns and adjustment to university: mediating role of coping strategies. J Family Res.

[49] Rahimi M, Meratian N, Zareei Mahmoodabadi H (2018). The role of family communication dimensions in adolescents’ depression with the mediation of cognitive flexibility. J Fundamentals Mental Health.

[50] Ajeli Lahiji L, Besharat MA (2019). The role of personality traits in predicting family functioning and quality of life among nurses, Shiraz, Iran 2017–2018. J Occup Health Epidemiol.

[51] Racic M, Todorovic R, Ivkovic N, Masic S, Joksimovic B, Kulic M (2017). Self- perceived stress in relation to anxiety, depression and health-related quality of life among health professions students: a cross-sectional study from Bosnia and Herzegovina. Zdr Varst.

[52] Gilliam ML, Berlin A, Kozloski M, Hernandez M, Grundy M (2007). Interpersonal and personal factors influencing sexual debut among mexican-american young women in the United States. J Adolesc Health.

[53] Lehrer JA, Shrier LA, Gortmaker S, Buka S (2006). Depressive symptoms as a longitudinal predictor of sexual risk behaviors among US middle and high school students. Pediatrics.

[54] Lundberg P, Rukundo G, Ashaba S, Thorson A, Allebeck P, Ostergren PO, Cantor-Graae E (2011). Poor mental health and sexual risk behaviours in Uganda: a cross-sectional population-based study. BMC Public Health.

[55] Giovenco D, Kahn K, Hughes JP, MacPhail C, Wagner R, Pettifor A (2020). Self-esteem as an Indicator of Transactional Sex among Young Women in Rural South Africa (HPTN 068). AIDS Behav.

[56] Duchesne AP, Dion J, Lalande D, Bégin C, Émond C, Lalande G (2017). Body dissatisfaction and psychological distress in adolescents: is self-esteem a mediator?. J Health Psychol.

[57] Ganesan S, Ravishankar SL, Ramalingam S (2018). Are body image issues affecting our adolescents? A cross-sectional study among college going adolescent girls. Indian J Community Med.

[58] Lin H-C, Lin Y-C (2018). The study of body image, self-esteem and sexual satisfaction of college students in southern Taiwan. Univers J Educational Res.

[59] Zare N, Daneshpajooh F, Amini M, Razeghi M, Fallahzadeh MH (2007). The relationship between self-esteem, general health and academic achievement in students of Shiraz University of Medical Sciences. Iran J Med Educ.

[60] Arshad M, Zaidi SM, Mahmood D (2015). Self-esteem & academic performance among university students. J Educ Pract.

[61] Niemz K, Griffiths M, Banyard P (2005). Prevalence of pathological internet use among university students and correlations with self-esteem, the General Health Questionnaire (GHQ), and disinhibition. Cyberpsychol Behav.

