# Supplementary material for: Factors affecting students’ attitudes towards reproductive health in the north of Iran: Designing an educational program
Source: BMC Public Health. 2023 Aug 16;23:1557. doi: 10.1186/s12889-023-16217-2 (PMC10428581; doi:10.1186/s12889-023-16217-2)
Supplement: Supplementary file 1 — Additional file 1. Data collection instruments. [file 12889_2023_16217_MOESM1_ESM.docx]

**Data collection instruments**

**RH Attitude Questionnaire:** Due to the non-availability of a relevant local standard questionnaire at the time of the study, a researcher-made questionnaire was used. By reviewing articles related to reproductive health including the RH questionnaire of the World Health Organization [22, 23], a 19-question questionnaire was first prepared. The questionnaire measured the attitude towards the relationship between boys and girls, methods of preventing sexually transmitted diseases, the use of contraceptive methods, teaching methods of preventing pregnancy and sexually transmitted diseases to young singles, and the RH needs of young people. Answers were determined based on a 5-point Likert scale with a maximum of five and a minimum of one.

Face and content validity were used to determine validity, and Cronbach's alpha coefficient was used to determine reliability to evaluate internal consistency. A qualitative method was used to evaluate face validity and qualitative and quantitative methods were used to evaluate content validity. In the quantitative evaluation of content validity, values higher than 0.59 in the Content validity index (CVI) were accepted. Also, the acceptance of items based on the content validity ratio (CVR) score was higher than 0.79. After validation, three questions were removed and finally, 16 questions remained with a score range of 16-80. Cronbach's standardized alpha reliability coefficient was 0.890. More details were published earlier [20].

**Interpersonal Communication Skills Test:** It has 19 five-choice questions with a score range of 19-95. A score below 45 means an acute communication problem, a score of 46-65 means a communication problem and 66-95 means a person's ability. Its validity and reliability have been confirmed with Cronbach's alpha of 0.73 in Iran [24].

**Revised Family Communication Pattern (RFCP) questionnaire:** It has a five-point scale. The questionnaire has two subscales, including dialogue orientation and alignment orientation. The range of scores in the subscale of dialogue is 0-60 and in the subscale of alignment is 0-44. Families that create a free and comfortable space for family members to participate in various topics have a dialogue pattern. The children of these families have high mental health and academic success and are more adaptable. In the alignment pattern, a higher score means aligning opinions and attitudes and avoiding conflict and dependence of members on each other. Communication in these families reflects obedience to parents and adults. Children in these families have low mental, physical and social health. The validity and reliability of the questionnaire with Cronbach's alpha coefficient of 0.87 for dialogue orientation and 0.81 was confirmed for alignment orientation in Iran [25].

**Depression Anxiety Stress Scale (DASS):** The short form of this scale has 21 items. Each of the constructs of depression, stress and anxiety is evaluated by seven different expressions. The range of answers is from never to always with a score of 0-3. Cronbach's alpha coefficient was reported as 0.93, 0.79, and 0.91 for depression, anxiety, and stress, respectively, and a test-retest reliability 0.74-0.88 in Iran. A score below 9 means normal, 10-13 is mild, 14-20 is Moderate, 21-27 is Sever, and above 27 is very severe depression. A score below 8 means normal, 8-9 is mild, 10-14 is Moderate and 15-19 is severe, and above 19 is very sever anxiety. A score below 15 means normal, 15-18 is mild, 19-25 is Moderate,26-33 is sever, and above 33 is very severe stress [26].

**Body Image Acceptance and Action Questionnaire (BI-AAQ):** The responses are scored on a 7-point Likert scale from not at all true to always true (score range 1-7). The minimum and maximum score is 12-84. A score below 19 means quite good, 19-27 is good, 28-42 is medium, and above 42 is weak body self-image. Its validity and reliability were confirmed in Iran. Higher scores indicate a lower self-image. Cronbach's alpha of 0.87 was reported for the questionnaire in Iran. Also, the retest coefficient of the tool was calculated as 0.72 [27].

**Rosenberg Self-Esteem Questionnaire:** It contains 10 statements according to a five-option Likert scale. The score range is 0 to 30. A score below 14 means unfavorable self-esteem, 15-25 is good, and above 25 is very good self-esteem. Its validity and reliability were confirmed in Iran [28].
