# Supplementary material for: Factors affecting students’ attitudes towards reproductive health in the north of Iran: Designing an educational program
Source: BMC Public Health. 2023 Aug 16;23:1557. doi: 10.1186/s12889-023-16217-2 (PMC10428581; doi:10.1186/s12889-023-16217-2)
Supplement: Supplementary file 2 — Additional file 2. Appendix. [file 12889_2023_16217_MOESM2_ESM.docx]

**Appendix**

**The content of the educational booklet on controlling stress, anxiety and the importance of the conversation orientation in the family communication pattern**

| **Educational content** | **Description** |
| --- | --- |
| **Stress** | |
| What is stress? |  |
| Stressor |  |
| Reaction to stress |  |
| Types of work stress | practical exercise sheet |
| stress symptoms | Physical, psychological and behavioral symptoms |
| Effects of stress |  |
| Prevention of stress | 1. Life events and stress  2. Daily troubles and chronic stress,  3. Success analysis  4. Nutrition and stress  5. Noise and stress  6. Sufficient rest and sleep  7. Time management  **Time management includes**:  a. Record daily schedule  b. Setting goals  J. prioritization  d. timing  **Important points to increase success in time management:**  * Maximize rewards  *Saying no  *Delegating tasks  * Limit interruptions |
| The importance of coping with stress |  |
| Healthy coping and unhealthy coping |  |
| Emotional confrontation |  |
| Relaxation techniques | 1- Deep breathing  2- Progressive muscle relaxation  3- mental imagery  4. Sports  5. Self-talk  Practice |
| Religion and spirituality |  |
| Problem-oriented coping | 1-Accepting the problem  2- Definition of the problem  3- Finding different solutions  4- Evaluating solutions and choosing the best solution  5- Implementing the solution and evaluating its effectiveness |
| A practical plan for healthy and effective coping with stress | **Step 1 -** Think about some reasons for learning to cope effectively with stress  **Step 2**- know your stress  **Step 3** - Know the signs of stress  **Step 4**- Reduce tension and negative emotions caused by stress  **Step 5** - Solve the problem that caused the stress  **Step 6** - Stay committed to your goals |
| Emotional coping methods | *Deep breathing  * Muscle relaxation  * imagination  * Sport  * Positive self-talk  * Religion and spirituality  * Imagining a pleasant future  * Nutrition  * Sufficient rest and sleep  * Planning to do favorite things  * Putting the situation in perspective  * Expression of feelings and concerns  * Imagining the future |
| Coping with stress worksheet |  |
| **Anxiety** | |
| What is anxiety? |  |
| The relationship between stress and anxiety |  |
| What is fear? |  |
| Normal fears |  |
| Abnormal fears |  |
| The relationship between fear and anxiety |  |
| Fears of adolescence and youth | A- Fear due to life problems  B- Fear of separation from parents  C. School fear  D. Fear in relation to sex  E- Fear of not being accepted in the peer group  F-Fear of committing things against religion and morals  G-Pathological fear |
| Signs and symptoms of anxiety |  |
| The nature and manner of anxiety |  |
| Types of anxiety | 1- Natural and normal anxiety  2- Abnormal anxieties  Causes and factors of anxiety |
| Natural and normal anxiety |  |
| Abnormal anxieties |  |
| Causes and factors of anxiety | 1- A feeling of loneliness  2- The sense of meaninglessness and aimlessness of life  3- Having a horrible picture of the face of death  4- Annoying pessimism  5- Fear of the future  6- Guilt and shame  7- Turning to a luxurious life  8- Dysfunctional attitudes and anxiety |
| Effective factors in reducing anxiety |  |
| Anxiety management methods | - Setting goals and solving problems  - Ways to deal with anxiety:  1. The coping skill of recognizing the symptoms of anxiety  2. The coping skill of avoiding unnecessary punishment and self-blame  3. The opposite skills of body control for self-soothing  4. Coping skill of controlling diet and exercise |
| Setting goals and solving problems |  |
| **Family communication pattern** | |
| Family communication pattern | |
| Conversation Orientation | |
| Conformity Orientation | |
| Family communication patterns | |
| Consensual family | |
| Pluralistic family | |
| Protective family | |
| laissez-faire family | |
| Explain communication patterns | |
